# If We Build It Comparative Psychologists Will Come. Commentary: A Crisis in Comparative Psychology: Where Have All the Undergraduates Gone?

**DOI:** 10.3389/fpsyg.2015.01700

**Published:** 2015-11-12

**Authors:** Jennifer Vonk, Eric Hoffmaster, Zoe Johnson-Ulrich, Silvia Oriani

**Affiliations:** Psychology, Oakland UniversityRochester, MI, USA

**Keywords:** comparative psychology, undergraduates, resources, access, careers, diverse, research

The title and tone of Abramson's (2015) opinion piece suggests a true crisis for the future of comparative psychology. We are not convinced that the situation is quite as dire. Furthermore, we question Abramson's concern with the integration of comparative psychology into other related fields, such as comparative cognition and evolutionary psychology. We agree that comparative psychology has suffered diminishing stature in the last several decades. However, we recognize that lack of engagement with the field stems at least in part from the following related issues neglected by Abramson; a serious lack of financial support for programs of research in comparative psychology, difficulty accessing subject populations, and changing norms regarding the ethical use of animal subjects in research. These issues have impacted the opportunities for researchers to make the kinds of significant contributions that inspired young scientists in the early part of this century.

In addition, we question whether comparative psychology needs to remain a distinct sub-field rather than allowing students to explore related topics from under the broad umbrella of other disciplines, such as animal behavior, zoology, ethology, behavioral ecology, comparative cognition, and evolutionary biology. Previously, Vonk and Shackelford (2012) argued that comparative psychology would be well served by integrating with evolutionary psychology. We argued that a multi-disciplinary and collaborative approach would move both fields substantially further forward than if they remained isolated. Greater innovation results from the amalgamation of insights unique to each area. This belief is consistent with recent endorsements from grant funding mechanisms, which specifically call for collaborative proposals. Collaborative programming is also strongly encouraged at the flagship conference put on by the American Psychological Association (APA). Collaborative graduate programs, such as “Brain, Behavior and Cognition” are becoming increasingly popular as well as they allow students to benefit from exposure to multiple perspectives and backgrounds. Furthermore, one of the truly important and unique contributions of comparative study is that it offers the ability to synthesize and evaluate findings from multiple species to provide greater insight with regard to underlying mechanisms. Animal research into concept formation and categorization can tell us how categories can be formed in the absence of language, which helps elucidate the relationship between language and abstraction in humans. Violation of expectation paradigms (Baillargeon, 2004; Heyes, 2014) have helped illuminate the distinction between implicit and explicit cognition, which applies both to preverbal human infants and non-human animals. Experiments with animal subjects cause us to question assumptions about human cognition and our place in the evolutionary process. There is no question that comparisons between species are illuminating but they can be made within various sub-fields, such as evolutionary, cognitive, and developmental psychology. From our point of view, as long as students are engaged in an area of study that can be defined as comparative, we have not “lost” undergraduates but have potentially provided many more unique paths to the same future goals. However, we share with Abramson a legitimate concern that students may find it difficult to identify courses and programs listed under disparate topics and titles. Young scientists should be encouraged to join organizations such as the Society for Behavioral Neuroscience and Comparative Cognition (Division 6 of APA) where a database containing information about such programs could be provided.

In order to emphasize the importance of the field, we must continue to support comparative psychologists so that their contributions can be realized and publicized. Departments must invest in resources and build collaborations with other institutions in order to provide the financial support and access to animal subjects that is required for junior scientists to build prolific programs of research. Abramson correctly notes that comparative psychology is not presented to undergraduates in a way that excites them. Whereas it is true that studying other species often allows us the opportunity to investigate important topics that would be impossible to study in humans (e.g., environmental vs. genetic influences by way of cross fostering studies, Holmes et al., 2005), this may not be the most palatable introduction to comparative psychology for the novice. Yet, this is the primary way the topic is introduced in introductory psychology texts according to Demarest (1987). Part of this problem is due to the paucity of well-trained comparative psychologists teaching introductory psychology courses. Most comparative psychologists find homes in teaching colleges where they do not have the resources to continue active programs of research. Fewer universities are able to house animals given restricted budgets and new welfare guidelines, which means that the best comparative investigators will be attracted only to top tier universities and may be teaching only graduate level courses. More and more of us rely on zoo, sanctuary, or shelter populations, but we lose the latitude to investigate the truly critical theoretical issues still plaguing our field. Furthermore, students no longer have direct access to interacting with animals in laboratory courses. Instructors have begun incorporating training sessions with canine labs and zoo trips so that students still have the opportunity to get valuable hands-on experience. These kinds of experiences are integral to exciting students about the kind of research they can conduct as comparative psychologists.

Perhaps we need to start the campaign for comparative psychology even earlier. It may be advantageous to work with the public education system to incorporate comparative psychology into the curriculum of advanced high school placement courses. In 2014, 259,789 (College Board, 2014) students took the AP psychology exam. Students who do well on the exam are then often able to place out of psychology 100 courses in college, which means the AP exam is the only exposure they'll receive to psychology. Thus, we need to make sure comparative content is represented before students even get to college.

It is also true that many promising young comparative psychologists end up moving into other areas, such as developmental and cognitive psychology, where research subjects are more accessible and affordable. If comparative psychologists are to remain engaged in the field, students must also be made aware of job possibilities outside of academia. We must ensure that students are trained to move seamlessly into organizations, such as zoos and welfare institutions, with an understanding of their missions and business models. We must extend our coverage of ethics in Psychology to ensure that students are well broached on the topic of animal ethics. Perhaps Ethics and Welfare should constitute its own introductory course in Psychology, rather than being subsumed within Research Methods. Until there is a clear future for the field, we would question whether it is morally responsible to encourage undergrads to pursue this field of inquiry. Without the promise of career opportunities in the field, we fear that any other measures to increase involvement in this area will simply contribute to the unemployment of educated students.

In sum, we agree that comparative psychology is worth preserving but we question the need to keep it separate from other related topics of study, and we emphasize a need to support junior scientists to become the valued researchers and instructors that will inspire others to follow in their footsteps.

## Author contributions

Each author contributed ideas to the manuscript, which was ultimately synthesized by the first author. All authors read and edited a draft of the final MS.

### Conflict of interest statement

The authors declare that the research was conducted in the absence of any commercial or financial relationships that could be construed as a potential conflict of interest.
